# Efficient Object Detection in Compressed Domain by Exploiting Knowledge Distillation from Pixel Domain

**DOI:** 10.3390/jimaging12070325

**Published:** 2026-07-18

**Authors:** Serhat Dikyar, Behcet Ugur Toreyin

**Affiliations:** 1 ASELSAN Inc., Ankara 06370, Türkiye; 2 Signal Processing for Computational Intelligence Research Group (SP4CING), İstanbul Technical University, İstanbul 34469, Türkiye; toreyin@itu.edu.tr; 3 Department of Electronics and Communication Engineering, İstanbul Technical University, İstanbul 34469, Türkiye; 4 Department of Artificial Intelligence and Data Engineering, İstanbul Technical University, İstanbul 34469, Türkiye

**Keywords:** compressed domain, knowledge distillation, efficient object detection

## Abstract

The proliferation of high-definition video data necessitates highly efficient processing pipelines for real-time edge analytics. However, traditional object detection architectures rely exclusively on pixel-domain inputs, which renders the computationally prohibitive decoding phase a latency bottleneck. In this paper, we propose a novel dual-phase framework designed to achieve fast and efficient object detection directly within the partially decoded compressed-domain data. First, we introduce a partial decoding paradigm featuring the Low-Frequency Spectral Prioritization method on the encoder side. By systematically discarding high-frequency residual coefficients and retaining only a sparse subset of fundamental spatial frequencies, this method dramatically reduces transmission payloads and accelerates the standard decoding process. Second, to recover the structural fidelity lost due to the intentional omission of residual data, we employ a multi-granularity cross-domain knowledge distillation architecture. This strategy aligns global contextual features, foreground boundary attention maps, and final response logits, transferring rich representational capacities from a high-performing pixel-domain teacher network to a lightweight compressed-domain student network. Comprehensive experiments utilizing RetinaNet, FCOS, and GFL object detection networks on the COCO-mini dataset demonstrate the superiority of the proposed framework. By retaining fundamental residual coefficients within the HEVC pipeline, the proposed method reduces average decoding latency while improving the mAP score by +0.96% over the conventional fully decoded pixel-domain baseline on the COCO-mini dataset.

## 1. Introduction

The proliferation of high-definition video data, driven by the ubiquity of surveillance systems, autonomous vehicles, and smart edge devices, has precipitated a fundamental challenge in computer vision: facilitating the storage and transmission of massive volumes of high-resolution media. To mitigate these bottlenecks, video is almost exclusively stored and transmitted in highly compressed formats, such as Advanced Video Coding (H.264/AVC) [1], High-Efficiency Video Coding (H.265/HEVC) [2], and the emerging Versatile Video Coding (VVC) [3]. These standard video codecs rely on complex mathematical transformations to eliminate spatial and temporal redundancies [4]. Consequently, extracting actionable semantic information directly from these compressed streams before full decoding has emerged as an efficient and viable paradigm for intelligent systems.

Specifically, standard deep learning pipelines are engineered to operate exclusively within the uncompressed pixel domain. This architectural assumption forces a sequential workflow where incoming bitstreams must undergo a complete decoding process before any semantic inference can occur. The standard decoding pipeline involves entropy decoding, inverse quantization, inverse spatial transformations, and complex in-loop filtering. These operations are profoundly resource-intensive. For instance, bypassing this decoding overhead by operating directly on compressed bitstreams can yield dramatic computational reductions. Recent evaluations on public action recognition datasets in [5] demonstrate that eliminating this full pixel reconstruction can result in up to 98% energy savings and a 55% gain in inference speed compared to established raw-domain models. Moreover, the reconstruction of high-frequency textural details, governed by residual data, constitutes a massive computational load, considering that residuals encompass up to 85–90% of the total data within intra-coded frames [6]. For resource-constrained edge devices, this intensive pixel-level reconstruction can create an extra latency bottleneck. Additionally, strictly minimizing the data utilized for video understanding aligns with modern privacy regulations that mandate data minimization [7].

To alleviate this computational bottleneck, recent studies have actively explored compressed-domain video analysis. This paradigm partially or entirely bypasses the computationally prohibitive transformations and calculation stages. Therefore, the compressed-domain approach has demonstrated significant latency reductions across a variety of visual tasks. While these approaches often employ partial decoding to retrieve intermediate representations rather than operating exclusively on raw entropy-coded bitstreams, they are broadly categorized as compressed-domain techniques. Such techniques are applied across diverse and complex visual tasks, e.g., spatiotemporal saliency detection [8], temporal sentence grounding [9], and video question answering [10]. In these studies, bypassing the full pixel-level reconstruction pipeline to leverage partially decoded codec elements is explicitly defined as compressed-domain analysis. Furthermore, the fundamental motivation for transitioning object detection to the compressed domain stems from the severe operational and infrastructural constraints inherent to modern real-time video analytics. While contemporary deep learning architectures deliver high detection precision, they demand immense processing power, creating a critical deployment dilemma. Deploying high-capacity processors directly at the data source drastically escalates edge hardware costs.

Conversely, transmitting heavy, high-resolution video streams incurs prohibitive bandwidth expenses and introduces network latency and disconnection risks. As demonstrated by recent scalable edge–cloud frameworks, extracting compressed-domain features directly at the edge can drastically reduce transmission payloads and cloud computational loads by filtering data before full decompression is ever required [11]. Additionally, traditional pixel-domain methods suffer from extensive decoding delays, establishing a major computational bottleneck, especially during the processing of intra-coded frames [12]. Beyond addressing these computational and transmission inefficiencies, executing semantic inference on partially decoded bitstreams inherently supports modern data minimization principles and secure video analytics. This execution thereby facilitating compliance with privacy frameworks such as the General Data Protection Regulation (GDPR) [13].

Despite the immense computational advantages of operating within the compressed domain, the intentional omission of residual data or the reliance on coarse structural syntax elements deprives neural networks of the fine-grained textural details necessary for robust semantic feature extraction. Consequently this leads to substantial degradation in detection accuracy. To mitigate this semantic loss without sacrificing the speed and bandwidth benefits of the compressed domain, knowledge distillation has emerged as a highly effective architectural strategy. At its core, knowledge distillation is a fundamental technique that transfers the representational capacity of a cumbersome, high-performing teacher model to a more compact or constrained student network, and it is widely employed in object detection [14,15].

In the context of compressed-domain analysis, this concept has been successfully adapted to bridge representational gaps across different data modalities. By transferring knowledge directly from the pixel domain, networks can effectively compensate for the lack of natural inductive biases inherent to compressed-domain representations [16]. Specifically, in this type of application, cross-modality distillation can be utilized to align the rich, robust feature space of a fully decoded pixel-domain teacher with the sparse, noisy inputs of a compressed-domain student. To handle structural artifacts, distillation architectures are often employed to allow the student to explicitly learn structural cues and hallucinate missing high-frequency textures caused by the omission of residual data. By matching intermediate feature maps and final detection responses across these two domains during the training phase, the student network implicitly learns to correct deformation artifacts. This allows the network to recover much of the detection precision lost to compression while maintaining the minimal data footprint required for real-time edge analytics [17,18].

In this study, we propose a dual-phase framework designed to achieve fast and efficient object detection in the compressed domain. We introduce a partial decoding paradigm that effectively reconciles the strict latency constraints of edge computing with the high semantic fidelity required for accurate perception. To achieve this, a selective residual removal method is implemented at the encoder side to systematically discard high-frequency coefficients, generating a highly compact bitstream that drastically accelerates the standard intra-decoding process. To recover the spatial accuracy lost due to the intentional omission of this residual data, we employ a multi-granularity cross-domain knowledge distillation strategy. This strategy transfers rich representational capacities from a high-performing pixel-domain teacher network to a lightweight compressed-domain student network by explicitly aligning features and response logits. While the extensive experimental evaluations in this research are conducted utilizing HEVC, the fundamental principles of selective spectral truncation and cross-modality distillation proposed herein are inherently adaptable and can be seamlessly extended to other legacy and emerging video codecs. Our comprehensive experiments utilizing RetinaNet, FCOS, and GFL architectures on the COCO-mini dataset demonstrate that retaining a sparse subset of fundamental residual coefficients effectively reduces average decoding latency, while the distillation-enhanced student networks achieve overall mAP scores that surpass their fully decoded, pixel-domain baselines. The contributions of this paper are summarized as follows:

- We propose a novel framework for fast and efficient object detection within partially decoded compressed-domain data with cross-domain knowledge distillation.
- We introduce an efficient partial decoding method driven by a simple yet effective Low-Frequency Spectral Prioritization (LFSP) strategy at the encoder. By short-circuiting bitstream generation post-quantization to retain only fundamental spatial frequencies, LFSP decreases bitstream size and decoding latency while preserving semantic fidelity without modifying the standard decoder.
- We formulate and propose a cross-domain knowledge distillation architecture to transfer representational capacity from a pixel-domain teacher to a compressed-domain student.

The rest of the paper is organized as follows: Section 2 provides a comprehensive review of related works concerning object detection and knowledge distillation methodologies within the compressed domain. Section 3 outlines the fundamental architecture of the High-Efficiency Video Coding standard, focusing specifically on spatial prediction and the standard residual calculation pipeline. Section 4 details the proposed methodology, including the modified partial decoding pipeline, the selective residual removal strategies, and the complete cross-domain knowledge distillation architecture. Section 5 presents the experimental setup, with quantitative results detailing the computational efficiency of the partial decode mechanisms. It also provides end-to-end detection performance evaluations across multiple object detection networks, and a comprehensive ablation study of the distillation methods. Finally, Section 6 concludes the paper with a summary of our primary findings.

## 2. Related Works

In this section, we provide a brief review of the existing literature that motivates the proposed framework. First, we explore recent advancements in pixel-domain object detection to establish the current state of the art and its inherent computational limitations. Second, we examine object detection methodologies within the compressed domain and explain how encoded syntax elements have been leveraged to bypass these bottlenecks. Finally, we explore the application of knowledge distillation methodologies, in which feature-level transfer and model compression techniques are utilized across the compressed domain. By synthesizing the progress and inherent limitations within these intersecting areas, we establish the theoretical motivation and practical necessity for our integrated approach.

### 2.1. Pixel-Domain Object Detection Methods

Recent years have witnessed remarkable progress in pixel-domain object detection, driven by the evolution of both deep convolutional networks and vision transformers. Notably, transformer-based architectures have emerged as highly effective solutions for small object detection, leveraging global attention mechanisms to overcome the spatial feature degradation typically experienced in conventional networks [19]. To address the specific challenges of detecting minute targets in unmanned aerial vehicle imagery, highly optimized architectures have also been developed. For example, enhancements to the YOLO framework, such as the integration of content-aware reassembly and interaction feature pyramid networks, specifically preserve fine-grained spatial details while maintaining the lightweight footprint required for onboard processing [20,21].

Beyond architectural modifications, researchers have devised specialized frameworks to handle extreme scale variations and environmental disturbances inherent to raw pixel data. In high-resolution aerial imagery, region-search methodologies utilize clustering algorithms, such as Gaussian mixture models, to isolate and crop focal regions containing dense object clusters, effectively normalizing object scales before detection [22,23]. Similarly, detecting small, salient objects under severely degraded visibility, such as low-light nighttime scenes, has been tackled by utilizing illumination and edge-driven networks that explicitly extract illumination-related features to guide saliency detection in non-uniform environments [24,25].

Furthermore, pixel-domain analysis has advanced to detect seamlessly camouflaged targets through concealed object detection networks. While possessing powerful detection capabilities, the heavy reliance of these models on background texture and global contextual cues makes them highly vulnerable to context-aware adversarial attacks that subtly perturb target textures to disrupt differential information [26,27]. More importantly, a universal limitation across all these sophisticated pixel-domain methodologies, whether they rely on heavy vision transformers, iterative region search, or complex illumination decoders, is their absolute dependence on fully decoded, high-resolution RGB imagery. For resource-constrained edge devices and autonomous systems, the prerequisite of decoding heavy video bitstreams before applying these advanced pixel-domain detectors introduces a latency bottleneck. This fundamental limitation necessitates a paradigm shift toward directly analyzing encoded bitstreams, leading to the exploration of compressed-domain object detection and cross-modal knowledge distillation.

### 2.2. Object Detection in Compressed Domain

The computational bottleneck inherent in fully decoding high-resolution video streams for pixel-domain analysis has catalyzed significant interest in compressed-domain object detection. Research in this field can be broadly categorized into two paradigms: syntax-driven detection in traditional codecs and temporal feature propagation.

Early and contemporary methodologies circumvent pixel-domain reconstruction by directly harvesting embedded syntax elements such as motion vectors (MVs), block partition types and Discrete Cosine Transform (DCT) coefficients from standardized bitstreams. For instance, detection algorithms for H.264/AVC have been proposed to evaluate macroblock types and partition modes to assign motion probability weights, effectively segmenting foreground from background [28]. Extending this to the HEVC standard, a combination of MVs, Coding Unit (CU) sizes, and prediction modes can be utilized to segment objects and classify them into semantic categories using a bag-of-words model based on HEVC syntax [6]. Additional techniques integrate spatio-temporal median filtering and fuzzy clustering on MVs and DCT coefficients to isolate moving targets across both codecs [29]. Furthermore, specific applications such as vehicle classification [30] and license plate detection [7] have been proposed. In [30], instead of calculating computationally heavy residuals, authors injected random perturbations to form intra-frames. On the other hand, block partition and prediction unit-based intra-frames have been employed in [7]. Moreover for intra-coded streams, HEVC intra-features have been harnessed alongside an iterative residual restoration algorithm to achieve fast object detection [12]. Building upon these application-specific optimizations, recent advancements have integrated dense object detection directly into the encoding pipeline to prioritize specific regions by dynamically categorizing Coding Tree Units into multi-tiered Regions of Interest without inflating overall transmission bandwidth [31]. Another recent study has introduced adaptive selective encryption mechanisms directly into the entropy coding stage by encrypting specific syntax elements. This allows intelligent edge devices to execute object detection directly on the base semantic and structural layers to address emerging data privacy constraints [32].

A second prominent category of research capitalizes on the temporal predictive structures of video codecs by propagating deep neural network features across frames. Instead of repeatedly extracting features from raw pixels, these architectures process sparse keyframes (I-frames) and use the freely available MVs and residual errors to warp features to subsequent predictive frames (P-frames). Pioneering this approach, architectures like the Motion-aided Memory Network (MMNet) align and refine features across scales, running significantly faster than single-image detectors [33]. In the context of video object segmentation, simply warped features can be augmented with a residual supplement module and a spatial attention module to recover lost appearance information and filter distractions [34]. Similarly, frameworks utilizing MVs for fast feature warping can exploit residual maps to guide frame selection and spatial correction, drastically reducing redundant computations [35]. Table 1 provides a comprehensive summary of the aforementioned literature, which outlines the proposed methods, object detection subcategories, and cross-domain knowledge distillation usage.

### 2.3. Knowledge Distillation from Compressed Domain

While directly analyzing compressed bitstreams reduces computational overhead, the inherent quantization artifacts and missing spatial details often cause accuracy degradation. To mitigate this gap, knowledge distillation is widely utilized to transfer representational capacities from high-performing, pixel-domain teachers to lightweight, compressed-domain students. Researchers have primarily directed these distillation strategies toward approximating complex temporal motion dynamics and aligning heterogeneous spatial feature representations.

Addressing the first challenge of temporal dynamics, optical flow provides highly accurate motion cues for video tasks but requires prohibitive runtime computation. Consequently, many studies use knowledge distillation to force networks operating on computationally inexpensive compressed modalities (such as motion vectors and residuals) to mimic optical flow-based teacher networks. Early methods tackled this by directly initializing student parameters from a teacher network or transferring soft supervision [36]. This approach evolved into specialized architectures. For example, flow-distilled networks replace the optical flow stream with a joint motion vector and residual stream supervised by optical flow features [37]. In semantic segmentation, boundary-aware distillation networks transfer pixel-wise probability maps and long-range dependencies to correct deformed features propagated by block motion vectors [18]. Additionally, feature alignment strategies coupled with temporal trilinear pooling have been utilized to directly remedy the representational weakness of motion vectors [38]. Recent methods also employ self-adaptive distillation losses to mimic flow-based reference probabilities by evaluating multiple combinations of modalities [39].

Beyond approximating temporal motion cues, addressing the second challenge of bridging the fundamentally heterogeneous feature spaces of raw pixels and compressed syntax elements is critical for overall spatial accuracy. Researchers resolve this structural domain gap by explicitly minimizing intermediate feature discrepancies. For example, cross-modality distillation methods construct pseudo-decoders and temporal graphs to align spatial appearance features and temporal relations between raw and compressed domains [17]. Similarly, pixel-to-compressed-domain knowledge transfer techniques minimize the squared error between intermediate feature maps of a pixel-domain vision model and its adapted compressed-domain counterpart [40]. Related methods employ gate modules to select compressed channels, improving accuracy via mean squared error distillation of middle-layer features [41]. Interestingly, cross distillation has also been applied in reverse, transferring knowledge from the compressed domain to the pixel domain to mitigate data scarcity issues in privacy-constrained environments [42]. Table 2 provides a comprehensive summary of the aforementioned literature, which outlines the proposed methods, their targeted downstream tasks, and whether they employ partially decoded (PD) image-like frames.

A comparative analysis of Table 1 and Table 2 reveals significant potential for advancing general object detection tasks through the use of cross-domain knowledge distillation alongside partially decoded image-like frames. Consequently, the proposed method employs cross-domain knowledge distillation from pixel-domain data to enhance general object detection performance on compressed-domain partially decoded image-like frames.

## 3. High-Efficiency Video Coding (HEVC)

The High-Efficiency Video Coding (HEVC) standard was developed collaboratively by the ITU-T Video Coding Experts Group and the ISO/IEC Moving Picture Experts Group to provide significantly improved compression performance over its predecessors. The core video coding layer of HEVC utilizes a block-based hybrid coding approach, combining interpicture and intrapicture prediction with two-dimensional transform coding. To process high-resolution video content efficiently, HEVC introduces a flexible partitioning structure based on the Coding Tree Unit. Each picture is divided into Coding Tree Units, which can be recursively partitioned using a quadtree structure into smaller blocks known as Coding Units. At this Coding Unit level, a picture area is coded either using spatial (intrapicture) or temporal (interpicture) prediction. Therefore, the Coding Unit forms the fundamental processing block for signal prediction and subsequent residual coding [2]. Since our proposed method works on intra-frames, we will mostly focus on intra-prediction.

### Residual Calculation

In HEVC architecture, the reconstruction of a spatial domain Coding Unit is mathematically formulated as the combination of a prediction signal and a residual error signal. Formally, a Coding Unit (CU) in the spatial domain, denoted as ${\mathrm{CU}}_{\mathrm{original}}$, is reconstructed via the summation of a prediction signal and a residual error signal:

(1) $${\mathrm{CU}}_{\mathrm{original}}={\mathrm{CU}}_{\mathrm{predict}}+{\mathrm{CU}}_{\mathrm{residual}}$$

where ${\mathrm{CU}}_{\mathrm{predict}}$ represents the spatial or temporal estimate derived from intra-prediction modes, and ${\mathrm{CU}}_{\mathrm{residual}}$ represents the difference between the original signal and the prediction.

The prediction signal ${\mathrm{CU}}_{\mathrm{predict}}$ serves as a baseline spatial estimate of the block. For intra-picture prediction, this signal is derived from previously decoded boundary samples of spatially neighboring blocks. The HEVC achieves high prediction accuracy by supporting 33 distinct directional orientations, along with Planar (surface fitting) and DC (flat) prediction modes. Because these advanced prediction modes successfully capture fundamental shapes and contours, the prediction block inherently retains the bulk of the essential structural information of the scene. The residual signal ${\mathrm{CU}}_{\mathrm{residual}}$ represents the mathematical difference between the original input block and the generated prediction signal. The prediction block captures much of the signal’s structural and directional content, while the residual represents the remaining prediction error, which often includes fine textural details and high-frequency components not well modeled by the predictor.

**Figure 1:**
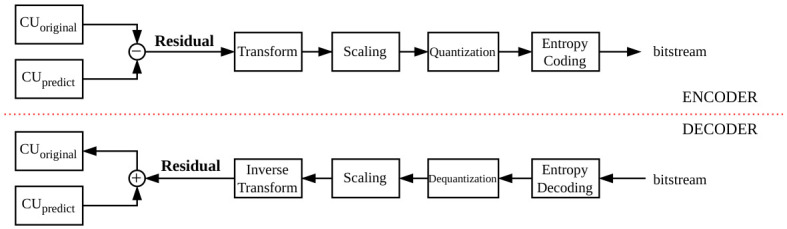
HEVC Residual Pipeline. The encoder computes a residual signal by subtracting the predicted block (${\mathrm{CU}}_{\mathrm{predict}}$) from the original (${\mathrm{CU}}_{\mathrm{original}}$), which is then transformed, scaled, quantized, and entropy-coded into a bitstream. In order to recover the original block, the decoder reverses this sequence, reconstructs the residual and adds it back to the prediction.

In the standard HEVC encoder pipeline, this spatial domain residual is transformed into the frequency domain using linear spatial transforms. HEVC employs integer basis functions that closely approximate the discrete cosine transform for block sizes ranging from 4 × 4 to 32 × 32, and alternatively utilizes a discrete cosine transform for 4 × 4 intrapicture predicted luma blocks. Following the transformation, the coefficients are scaled, quantized, and entropy coded into the final bitstream. To reconstruct the image at the decoder side, the system must reverse this pipeline. The decoder extracts the quantized transform coefficients via entropy decoding, applies inverse scaling (dequantization), and performs computationally heavy inverse spatial transformations to duplicate the residual signal. Finally, this reconstructed residual is added back to the prediction signal to form the decoded picture. The aforementioned HEVC residual pipeline is illustrated in Figure 1.

While reconstructing the full residual is indispensable for achieving high perceptual quality, the inverse transformation process introduces significant computational latency and overhead. Because the fundamental low-frequency structural information is predominantly preserved within the prediction signal and the lowest frequency residual coefficients, the exhaustive calculation and inverse transformation of all high-frequency residual components presents a notable computational bottleneck. Recognizing this mechanism is vital for developing compressed-domain acceleration strategies, where selectively bypassing the full inverse transform of these residuals can drastically reduce decoding time while maintaining sufficient semantic integrity for automated perception architectures.

## 4. Method

This section details the proposed framework for highly efficient, compressed-domain object detection. We first provide a high-level overview of the dual-phase architecture that includes offline model synthesis and online edge deployment. Furthermore, we introduce the modified HEVC pipeline and evaluate specific selective residual removal methodologies designed to accelerate decoding by isolating low-frequency structural data. Finally, we formulate the cross-domain knowledge distillation strategy that aligns multi-granularity features and logits to bridge the semantic gap between the pixel-domain teacher and the compressed-domain student.

### 4.1. The Method Overview

**Figure 2:**
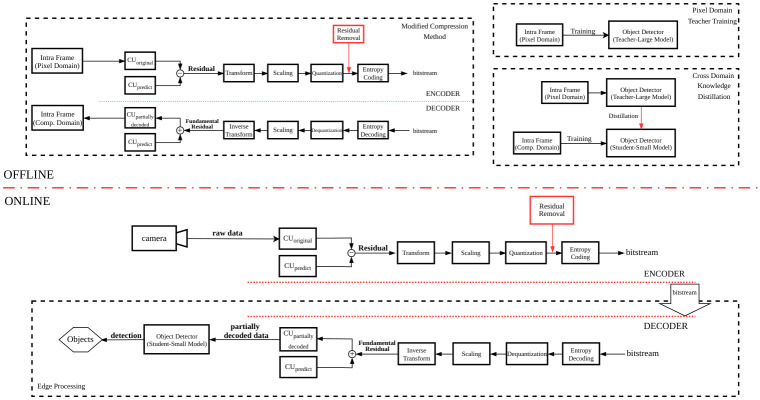
Diagram illustrates a dual-phase framework designed to achieve efficient object detection by processing data in the compressed domain. During the offline stage, a large teacher model transfers its sophisticated understanding of pixel-level images to a smaller, more efficient student model through knowledge distillation. Thanks to this distillation process, during the online stage the student model operates on partially decoded data at the edge and reduces the computational burden typically required for full image reconstruction. By eliminating unnecessary residual data and focusing solely on the essential information needed for accurate detection, the inference gets faster in resource-constrained environments.

The proposed framework can be explained in two folds. The first phase is Offline Development in which dataset synthesis and model training have been utilized. The second is Online Deployment Phase where the modified HEVC pipeline enables a fast inference on edge processing units. The overview of the proposed method is illustrated in Figure 2.

#### 4.1.1. Offline Development and Dataset Synthesis

The primary objective of the offline phase is to generate a specialized dataset and train a robust detection model. To achieve this, a standard pixel-domain intra-frame set or dataset is processed through a modified compression pipeline and the object detection network is trained with partially decoded data to handle visually degraded inputs.

In the modified compression, the intra-frames undergo standard prediction (${\mathrm{CU}}_{\mathrm{original}}$), transformation, and quantization on the encoder side. However, after quantization our critical intervention occurs and selective residual removal is implemented here. Because the encoder simply does not encode the explicitly zeroed coefficients, the resulting bitstream maintains bitstream-level compliance and remains HEVC-compliant. Therefore, we generate a compliant bitstream containing only the fundamental residuals required to maintain structural integrity by systematically discarding non-essential residual coefficients. This bitstream is then processed by a standard conforming decoder to create a compressed-domain dataset.

Moreover, a knowledge distillation strategy is applied to recover the accuracy lost due to compression artifacts. A high-capacity teacher is trained using the rich feature representations of the original pixel-domain frames. On the other hand, a lightweight student is trained on the compressed-domain frames, while the teacher network guides the student through a knowledge distillation process. Thanks to knowledge distillation, the student network infers missing semantic details from the partial decoded inputs. The complete procedure for this offline development and dataset synthesis is outlined in Algorithm 1.
**Algorithm 1** Offline Development and Dataset Synthesis**Require:** Dataset D of raw images $I_{\mathrm{raw}}$ and ground-truth labels Y, Teacher Model T, Student Model S**Ensure:**  Trained Student Model S optimized for the compressed domain1:**for** each $\left(I_{\mathrm{raw}},Y\right)$ of D **do**— 1. Partial Decode for Compressed Domain Dataset —2:    partiallyGeneratedBitstreams$\leftarrow \mathrm{modifiedEncoderWithResidualRemoval}(I_{\mathrm{raw}})$3:    framesInCompressedDomain←unmodifiedDecoder(partiallyGeneratedBitstreams)— 2. Pixel Domain Teacher Training —4:    $T_{\mathrm{pixel}}\leftarrow \mathrm{trainingInPixelDomain}(T,D$)— 3. Cross-Domain Knowledge Distillation —5:    $Feat_{T},Pred_{T}\leftarrow T_{\mathrm{pixel}}\left(D\right)$6:    $Feat_{S},Pred_{S}\leftarrow S\left(framesInCompressedDomain,Y\right)$7:    $S_{\mathrm{compressed}}\leftarrow \mathrm{trainingKnowledgeDistillation}\left(Feat_{T},Pred_{T},Feat_{S},Pred_{S}\right)$8:**end for**

#### 4.1.2. Online Deployment and Fast Inference

The practical deployment of this framework is addressed in the online phase. During operation, the camera captures raw data and the data is processed by the modified encoder where the selective removal of residuals occurs after quantization in the pipeline. Consequently, the encoding process is accelerated and significantly fewer bits are transmitted to the decoder.

At the perception unit, the original decoder reconstructs the image. Since the incoming bitstream has been effectively short-circuited to omit heavy residual data, the decoder produces a partially decoded image significantly faster than it would do for a fully conforming bitstream. The pre-trained student network then performs inference directly on this partially decoded data.

This trade-off could be particularly effective for robotics application such as autonomous driving scenarios. While the resulting images may lack the fine-grained texture required for human aesthetic viewing, they maintain high semantic fidelity for critical, large-scale targets. The result is a high-speed detection pipeline that operates within the strict capacity limits of automotive data networks while remaining reliable for safety-critical navigation. The complete procedure for this online deployment and fast inference is outlined in Algorithm 2.
**Algorithm 2** Online Deployment and Fast Inference**Require:**  Trained Student Model $S_{\mathrm{compressed}}$, Incoming raw data $I_{\mathrm{raw}}$ from camera**Ensure:**  Detected Objects— 1. Partial Decoding —1:partiallyGeneratedBitstreams$\leftarrow \mathrm{modifiedEncoderWithResidualRemoval}(I_{\mathrm{raw}})$2:framesInCompressedDomain←unmodifiedDecoder(partiallyGeneratedBitstreams)— 2. Object Detection Forward Pass —3:$Objects\leftarrow S_{\mathrm{compressed}}\left(framesInCompressedDomain\right)$4:**return** Objects

### 4.2. Modified HEVC Pipeline for Partial Decoding

**Figure 3:**
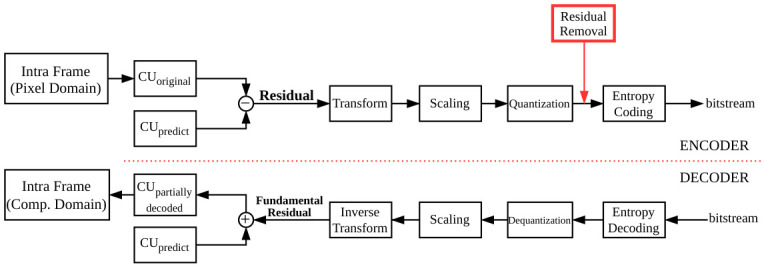
In the modified pipeline, the intra-frames undergo standard prediction, transformation, and quantization on the encoder side. However, after quantization our critical intervention occurs and selective residual removal is implemented here.

To address the latency and efficiency constraints inherent in object detection, we introduce a modified video coding pipeline that deviates from the standard HEVC architecture. This modification is premised on the observation that object detection algorithms generally prioritize low-frequency structural contours over the high-frequency textural fidelity required for human visual perception (Figure 3).

Standard HEVC reconstruction operates on a block-based hybrid architecture. We define the modified HEVC pipeline through the introduction of a spectral truncation function, Φ(·), which is applied after the quantization stage at the encoder. This function selectively filters the quantized residual coefficients $Q_{\mathrm{residual}}$ and generates a sparse subset of fundamental coefficients $Q_{\mathrm{residual}}^{\prime }$. Consequently, a partially decoded representation ${\mathrm{CU}}_{\mathrm{partially}\;\mathrm{decoded}}$ is reconstructed at the decoder with fundamental coefficients $Q_{\mathrm{residual}}^{\prime }$ as given below:

(2) $$Q_{\mathrm{residual}}^{\prime }=\Phi \left(Q_{\mathrm{residual}}\right)$$

(3) $${\mathrm{CU}}_{\mathrm{partially}\;\mathrm{decoded}}={\mathrm{CU}}_{\mathrm{predict}}+T^{-1}\left(Q_{\mathrm{residual}}^{\prime }\right)$$

where $T^{-1}$ denotes the inverse transform applied to the fundamental residuals.

The proposed framework introduces a distinct architectural deviation at the encoder side of the standard HEVC pipeline. On the other hand, the decoder side remains entirely unmodified. In the conventional pipeline, normally all non-zero quantized coefficients are passed directly to the entropy coding stage. In our modified approach, a residual removal module is inserted immediately post-quantization. This module systematically discards high-frequency residual coefficients before they enter the entropy encoder. By short-circuiting the bitstream generation process, the encoder deals with significantly less data, yielding a highly compact bitstream.

The method employs a standard HEVC decoder to reconstruct the image. A standard decoding process typically necessitates computationally expensive inverse transforms on a dense spectrum of coefficients. However, because the incoming bitstream has already been stripped of high-frequency residual data, the standard decoding process is naturally accelerated and highly efficient. The computationally demanding inverse transformations are executed strictly on a sparse subset of fundamental coefficients. Consequently, without any structural modifications to the decoder itself, the system rapidly generates a partially decoded representation by summing the prediction block ${\mathrm{CU}}_{\mathrm{predict}}$ with only the fundamental residuals.

The primary utility of this modified pipeline is the reconciliation of high-bitrate sensor data with the limited computational resources of edge computing devices. By bypassing the full inverse transform and other stages, the decoding process is significantly accelerated. Moreover, the selective removal of coefficients prior to entropy coding directly reduces the volume of data transmitted. This modification also lowers the bitrate requirements for network transmission. Furthermore, it retains the essential object integrity, although the resulting frames exhibit artifacts that degrade human perceptual quality. The removal of coefficients explicitly reconciles the disparity between the massive data volume of residual coefficients and the minimal number required for detection. Specifically, raw data volume and semantic information content can be effectively decoupled in this context, as a small number of low-frequency coefficients carry a disproportionate amount of the structural information necessary for machine vision tasks.

### 4.3. Selective Residual Removal Methodologies

A critical component of our framework is determining which quantized residual coefficients to retain. In order to achieve maximum semantic fidelity and minimum decoding time, we have evaluated and compared specific removal methodologies for selective residual removal:

**Simple Raster Sequence Prioritization (SRSP):** Serving as the standard baseline method for comparison, this approach employs a fixed spatial traversal strategy for coefficient selection, entirely ignoring the underlying intra-prediction characteristics and the spectral significance of the residuals. The complete formal algorithm for SRSP is outlined in Algorithm 3.

**Low-Frequency Spectral Prioritization (LFSP):** This methodology prioritizes the spectral distribution of the quantized residual signal by targeting specific frequency bands within the transformed block. Specifically, it explicitly selects only the upper-left quantized residual coefficients, which correspond to low-frequency components. Therefore, we retain the fundamental structural silhouettes essential for robust object recognition while effectively zeroing out high-frequency textural noise. Unlike previous compressed-domain methodologies that harvest quantized residual coefficients from fully or partially generated bitstreams for post-processing, clustering or complex coefficient retention selection methodologies, LFSP acts as a simple structural intervention directly at the encoder. By simply discarding high-frequency coefficients immediately post-quantization and before entropy coding, LFSP short-circuits bitstream generation. This produces a natively sparse, HEVC-compliant bitstream that intrinsically accelerates unmodified standard decoders. The complete formal algorithm for LFSP is outlined in Algorithm 4.

**Algorithm 3** Simple Raster Sequence Prioritization (SRSP)
**Require:**  Quantized transform coefficients *C*, Block dimensions W×H, Threshold of retained coefficients $N_{kept}$**Ensure:**  Retained coefficient array *C*
1:**if** block is Intra-coded **then**2:    TotalCoeffs←W×H3:    **for** $i=N_{kept}$ **to** TotalCoeffs−1 **do**4:        C[i]←05:    **end for**6:
**end if**
7:**return** *C*


**Algorithm 4** Low-Frequency Spectral Prioritization (LFSP)
**Require:**  Quantized transform coefficients *C*, Block dimensions W×H, Threshold of retained coefficients $N_{kept}$,**Ensure:**  Retained coefficient array *C*
1:

$N_{dim}\leftarrow \sqrt{N_{kept}}$

2:**if** block is Intra-coded **then**3:    **for** y=0 **to** H−1 **do**4:        **for** x=0 **to** W−1 **do**5:           **if** $x\geq N_{dim}$ **or** $y\geq N_{dim}$ **then**6:               C[y×W+x]←07:           **end if**8:        **end for**9:    **end for**10:
**end if**
11:**return** *C*


**Figure 4:**
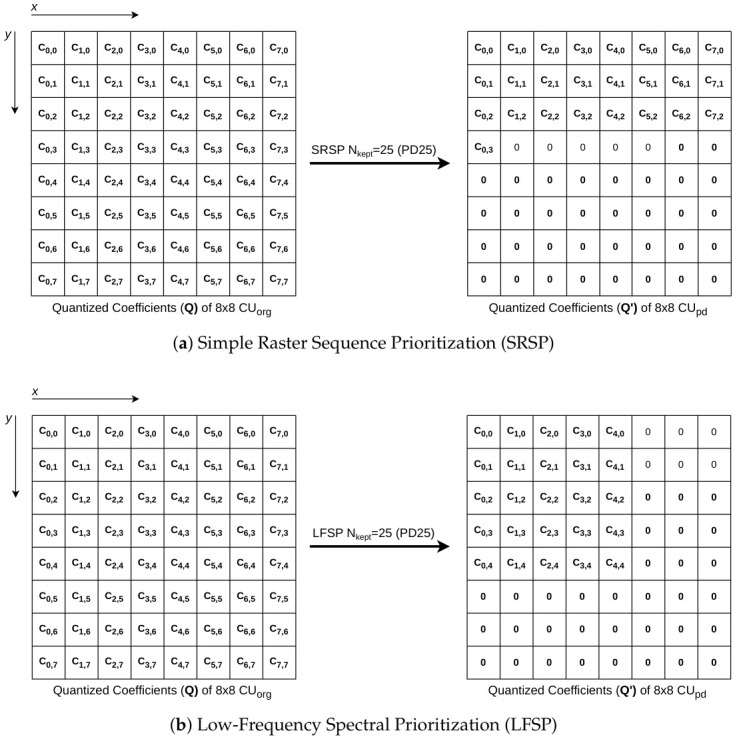
Selective Residual Removal Methods. The indices (*x*, *y*) are denoted as $c_{x,y}$ in the figure.

We propose the LFSP method as the optimal strategy for downstream machine vision tasks. Unlike the baseline approach, the LFSP configuration consistently yields a partially decoded representation that maintains vastly superior structural integrity. Figure 4 illustrates how quantized residual coefficients are retained and zeroed under each configuration for an exemplary 25 retained coefficients. Due to the fact that our chosen method is LFSP, which selectively retains the upper-left region of the CU, the candidate set for the number of retained coefficients specifically consists of perfect squares to evenly encapsulate both horizontal and vertical scans.

### 4.4. Cross-Domain Knowledge Distillation

We propose a cross-domain knowledge distillation method by using framework in [43] to transfer the robust representational capacity of a large-scale teacher network operating in the pixel domain to a compact student network operating in the compressed domain. The fundamental challenge addressed by this framework is the significant semantic gap between the two domains: while the teacher extracts features from spatially coherent pixel-domain data, the student must infer similar semantics from partially decoded compressed-domain data. To bridge this gap without incurring excessive computational overhead, we employ a multi-granularity distillation strategy that aligns representations at both the intermediate feature level and the final response level, namely Global Feature Distillation (GFD), Foreground–Background Feature Distillation (FBFD) and Logit-based Response Distillation (LRD). While our approach integrates with the general knowledge distillation framework proposed for the pixel domain, it introduces specialized cross-domain adaptations to resolve the unique artifacts of partial decoding.

**Figure 5:**
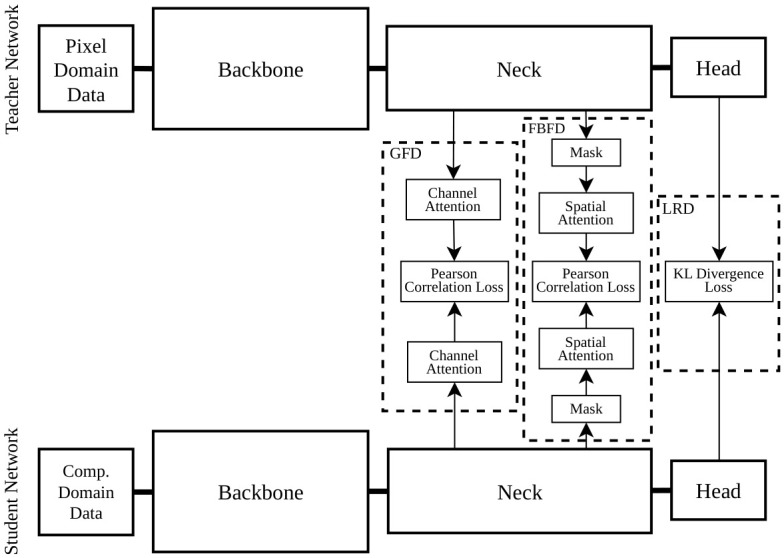
Knowledge Distillation Method. We employ a multi-granularity distillation strategy that aligns representations at both the intermediate feature level and the final response level, namely Global Feature Distillation (GFD), Foreground–Background Feature Distillation (FBFD) and Logit-based Response Distillation (LRD).

Standard modern object detectors generally follow a three-stage architecture: a backbone for initial feature extraction, a neck for multi-scale feature fusion, and a head for final classification and bounding box regression. The distillation framework integrates at the neck and head stages since the backbone features in the compressed domain are too domain-specific to directly align with pixel-domain backbone features. Cross-domain knowledge distillation architecture is depicted in Figure 5.

Prior to detailing the specific distillation objectives, we formalize the attention mapping functions utilized to extract statistical descriptors from the high-dimensional convolutional feature maps. Let $F\in R^{C\times H\times W}$ denote a feature tensor extracted from a specific network layer, where *C*, *H*, and *W* represent the channel, height, and width dimensions, respectively. To bridge the semantic gap between the pixel and compressed domains, we decouple these features into independent channel and spatial descriptors.

**Channel Attention ($\Psi_{c}$):** The channel attention function aggregates the spatial information of the feature maps to capture the global context and semantic distribution across the channel dimension. By performing global average pooling over the spatial dimensions, we project the tensor *F* into a channel descriptor vector $\Psi_{c}\left(F\right)\in R^{C}$. For the *c*-th channel, this operation is formulated as
  (4) $$\Psi_{c}{\left(F\right)}_{c}=\frac{1}{H\times W}\sum_{i=1}^{H}\sum_{j=1}^{W}F_{c,i,j}$$
  This mapping evaluates the global activation strength of each semantic filter. By isolating these channel-wise statistics, the function allows the distillation process to align the broad scene understanding between the teacher and student networks independently of localized spatial variations.
**Spatial Attention ($\Psi_{s}$):** Conversely, the spatial attention function collapses the channel dimension to highlight highly discriminative spatial regions. The tensor *F* is projected into a spatial activation map $\Psi_{s}\left(F\right)\in R^{H\times W}$ by calculating the mean absolute activation across all *C* channels at each spatial location (i,j):
  (5) $$\Psi_{s}{\left(F\right)}_{i,j}=\frac{1}{C}\sum_{c=1}^{C}\left|F_{c,i,j}\right|$$
  This mapping generates a two-dimensional spatial importance map that emphasizes foreground object characteristics. It is particularly critical for guiding the student network to focus on essential structural information while effectively ignoring background compression artifacts.

#### 4.4.1. Global Feature Distillation (GFD)

To capture the global context, we employ a channel attention mechanism. This transformation aggregates spatial information to produce a channel descriptor, effectively re-weighting the feature maps to emphasize semantically salient channels. Let $F_{T}$ and $F_{S}$ denote the feature maps of the teacher and student at a given neck level. The channel attention function $\Psi_{c}\left(\cdot \right)$ recalibrates these features to highlight which channels are contributing most to the global scene representation.

To quantify the similarity between these recalibrated representations, we utilize the scale-invariant Pearson Correlation Coefficient rather than Mean Squared Error (MSE). Unlike MSE, which rigidly penalizes absolute magnitude differences and struggles with the disparate feature scales across different domains, Pearson correlation centers the data and makes it robust to the inherent statistical differences between the pixel and compressed domains. The objective function is defined as

(6) $$L_{GFD}=1-\rho \left(\Psi_{c}\left(F_{T}\right),\Psi_{c}\left(F_{S}\right)\right)$$

where ρ(X,Y) is the Pearson Correlation Coefficient calculated over the flattened feature vectors. By maximizing this correlation, we ensure that the student learns the global semantic distribution of the scene understanding regardless of the domain-specific intensity variations.

#### 4.4.2. Foreground–Background Feature Distillation (FBFD)

This branch explicitly decouples the feature maps into foreground and background regions using a binary mask *M*, derived from the ground-truth bounding boxes. Within the foreground regions, we apply a spatial attention $\Psi_{s}$ mechanism. Unlike channel attention, spatial attention generates a spatial importance map, highlighting the most discriminative regions within the object boundaries.

Similar to the global branch, we employ the Pearson Correlation Coefficient for the loss function. This choice is critical: it forces the student to replicate the pattern of the teacher’s spatial activation rather than the exact pixel values. The loss is formulated as

(7) $$L_{FBFD}=1-\rho \left(\Psi_{s}\left(F_{T}\right)⊙M,\Psi_{s}\left(F_{S}\right)⊙M\right)$$

where ⊙ denotes element-wise multiplication. We assign a dominant weight to this component to accurately localize and define object boundaries against compression artifacts.

#### 4.4.3. Logit-Based Response Distillation (LRD)

Feature alignment does not guarantee identical decision logic. The teacher output logits, prior to the final prediction, contain soft probability distributions that encode inter-class similarities. We minimize the Kullback–Leibler (KL) Divergence between the teacher normalized logits $P_{T}$ and the student logits $P_{S}$:

(8) $$L_{LRD}=D_{KL}(P_{T}\left|\right|P_{S})=\sum_{i}P_{T}\left(i\right)\log\left(\frac{P_{T}\left(i\right)}{P_{S}\left(i\right)}\right)$$

where $D_{KL}$ denotes Kullback–Leibler Divergence to measure the statistical distance of student and teacher logits.

#### 4.4.4. Total Objective Function

The total training objective combines the standard detection loss $L_{det}$ with the proposed multi-level distillation losses. The total loss $L_{total}$ is summed across the relevant neck levels:

(9) $$L_{tot}=L_{det}+\gamma_{global}L_{GFD}+\gamma_{fbg}L_{FBFD}+\gamma_{logits}L_{LRD}$$

where $\gamma_{global}$, $\gamma_{fbg}$, and $\gamma_{logits}$ are empirically determined weighting hyperparameters that balance the contributions of the global feature, foreground–background feature, and logit-based response distillation losses, respectively. In the total loss function, the hierarchical formulation ensures a robust transfer of knowledge: GFD aligns the semantic channel distributions, FBFD sharpens the spatial focus on objects using correlation-based matching, and LRD refines the final classification logic.

## 5. Experiments

In this section, we present a comprehensive empirical evaluation of the proposed framework. Following an overview of the experimental setup, we first analyze the modified partial decoding pipeline to quantify the trade-offs between structural fidelity, bitrate, and latency, thereby establishing the optimal residual retention threshold. We subsequently evaluate the end-to-end object detection performance of our compressed-domain approach across multiple architectures, demonstrating its computational and accuracy advantages over standard fully decoded baselines. Finally, we conclude with an ablation study that isolates and rigorously quantifies the impact of the individual cross-domain knowledge distillation components.

### 5.1. Experimental Setup

The proposed efficient object detection method is evaluated using the complete COCO dataset [44] and the COCO-mini dataset [45], which is a statistically validated and curated subset of the COCO dataset. The purpose of specifically utilizing this dataset is to significantly reduce the computational cost and accelerate the turnaround time of our extensive knowledge distillation training/ablation experiments. The COCO-mini dataset comprises approximately 20% of the full training set, which is 25,000 training images containing around 184,000 object instances across the 80 distinct COCO categories. The dataset also includes 5000 validation images that are identical to the full set to benchmark the detection accuracy.

To ensure the dataset remains highly representative of the original data distribution, this subset was constructed to strictly preserve the overall and per-class ratios of small, medium, and large objects, alongside the proportion of object instances for each class. Furthermore, rigorous empirical validations across multiple state-of-the-art detectors demonstrate that models trained on COCO-mini exhibit a strong positive correlation with their counterparts trained on the complete dataset, proving its reliability as a highly representative empirical baseline [45].

The dataset images possess variable spatial resolutions and are originally stored in JPEG format. To process them for our framework, we convert all images to raw (YUV) format using ffmpeg, and then encode and decode them with the modified HEVC Reference Software HM 18.0 [46]. For the encoding process, the default *encoder_intra_main.cfg* configuration parameters are utilized. Specifically, we employ a Quantization Parameter (QP) of 32, a maximum coding unit (CU) size of 64×64 and an intra-period of 1 as defined by the standard main profile. All experiments and evaluations are conducted on an Intel Xeon Gold 6338 CPU and an Nvidia P100 GPU running the Rocky Linux 9.2 operating system.

### 5.2. Partial Decoding Experiments

In these experiments, we will present the qualitative and quantitative evaluation of the modified partial decoding pipeline of the established methodologies for selective residual removal. Our analysis focuses on the trade-offs between processing latency, bitstream economy and the resulting visual fidelity.

**Figure 6:**
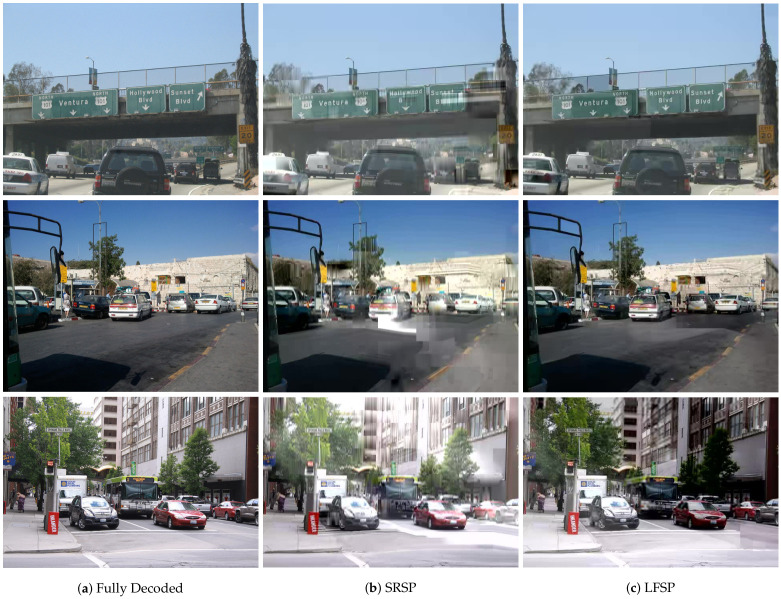
Qualitative comparison of partially decoded images generated by the SRSP (Simple Raster Sequence Prioritization) and LFSP (Low-Frequency Spectral Prioritization) residual removal methods with their corresponding fully decoded counterparts. Visually, LFSP successfully preserves the fundamental structures essential for object recognition, whereas the baseline SRSP method suffers from artifact degradation.

Qualitative analysis of the partially decoded images generated by both LFSP and SRSP is depicted in Figure 6. The sample images for different residual removal methods are both constructed by retaining exactly 64 quantized residual coefficients. Visually, the LFSP method demonstrates superior structural preservation compared to the baseline residual removal method, which is SRSP.

To establish a robust empirical baseline for quantitative analysis, the experimental methodology involved evaluating the proposed configurations on validation images of the dataset. Consequently, the performance metrics presented in Table 3 represent the average values computed across 5000 images. Within this evaluation, several key metrics are defined: decoding time denotes the duration required to decode the bitstream encoded with a given method; bitstream size indicates the volume of the resultant encoded bits generated by the modified encoder via the selective residual removal method; PSNR represents the Peak Signal-to-Noise Ratio of the decoded image; and SSIM measures the structural similarity between the original image and the decoded images. Quantitatively, both the SRSP and LFSP configurations demonstrate significant computational and bandwidth advantages over the full decoding. By selectively removing residual coefficients, the average bitstream size is approximately halved and average decoding latency is substantially reduced from 19.1 ms to 12.8 ms and 13.9 ms. Furthermore, because the encoder simply does not encode the zeroed coefficients, the average bitstream size decreases significantly from 18.46 Kb to 9.48 Kb and 11.25 Kb for both SRSP and LFSP, respectively. Consequently, this reduction in bitstream size leads to a corresponding decrease in decoding time.

While the SRSP method exhibits a slight improvement in speed and compression, the quality metrics (PSNR and SSIM) reveal a critical deficiency. Since SRSP employs a fixed spatial traversal strategy that entirely ignores the underlying prediction characteristics and the spectral significance of the residuals, severe degradation can be inferred from the quality metrics. In contrast, the LFSP configuration yields vastly superior structural integrity and achieves an average PSNR of 26.57 dB and an SSIM of 0.8491. Therefore, LFSP compared to SRSP represents a necessary and justified minor latency and bitstream size trade-off to ensure reliable object detection.

**Figure 7:**
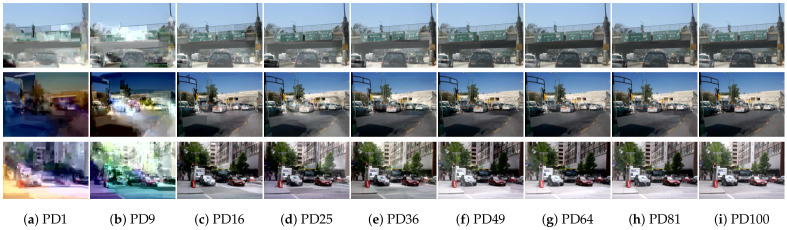
Qualitative evaluation of the LFSP method’s scalability across varying residual retention thresholds. The label PD*x* indicates a partially decoded image where exactly *x* quantized residual coefficients are retained (e.g., PD64 retains 64 coefficients). The visual fidelity of the reconstructed images consistently increases as more coefficients are preserved.

In order to investigate the scalability of the LFSP method, the subsequent experiments have been conducted by systematically varying the number of retained quantized residual coefficients. The quantitative result summary and qualitative results of partially decoded images are given in Table 4 and Figure 7, respectively.

**Figure 8:**
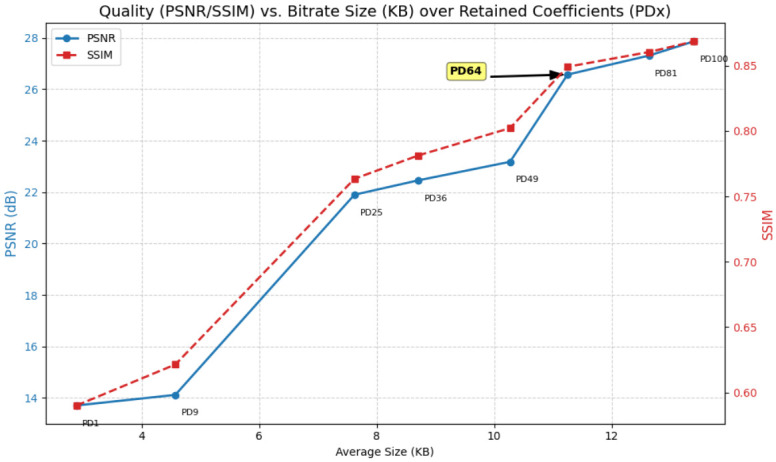
Performance scalability of the LFSP configuration over varying retained coefficients. The operational point PD64 demonstrates the optimal trade-off, achieving a steep increase in both PSNR and SSIM, beyond which the rate of visual quality improvement plateaus while bitrate size (Average Size) continues to increase.

As can be seen from the results, the visual quality and structural integrity of the reconstructed images consistently increase as the number of retained residual coefficients increases. However, this progression reveals a clear relationship of diminishing returns between computational latency, bitstream economy, and structural fidelity. While configurations utilizing fewer coefficients offer extreme compression and rapid decoding speeds, they struggle to reconstruct sufficient semantic detail. The configuration retaining exactly 64 quantized residual coefficients emerges as the optimal operational threshold. It achieves a substantial leap in visual fidelity with a PSNR of 26.57 dB and an SSIM of 0.8491, while maintaining a highly efficient decoding latency of 13.9 ms and a compact bitstream size of 11.25 KB. As illustrated in the performance curves (Figure 8), this configuration marks the steepest gain in both quality metrics; beyond PD64, the rate of improvement distinctly plateaus, resulting in diminishing returns.

The incorporation of additional high-frequency spectral data introduces diminishing returns beyond the 64-coefficient threshold. Expanding the retention to 81 and 100 coefficients incurs a steeper penalty in processing latency due to the heavier computational burden placed on the inverse transform and dequantization stages. Conversely, the corresponding gains in structural quality become highly marginal; PSNR and SSIM increase slightly. The negligible visual and objective quality improvements beyond 64 coefficients do not justify the disproportionate escalation in decoding time and transmission bandwidth. Consequently, we found that the 64-coefficient LFSP configuration is the optimal input representation.

### 5.3. Efficient Object Detection in Compressed Domain

To comprehensively evaluate the efficacy of the proposed method within a complete inference pipeline, we employed RetinaNet [47] object detector utilizing a lightweight ResNet-18 student backbone, guided by a high-capacity ResNet-50 teacher backbone. The experimental evaluation was implemented utilizing MMDetection [48] integrated with a knowledge distillation framework [43]. The input to the student network consists of highly quantized, partially decoded representations generated via the LFSP residual removal method.

To facilitate a clear interpretation of the experimental results, we first define the configuration nomenclature and metric headers utilized in our analysis and tables. Img Type denotes the specific input dataset configuration: PD*x* represents a Partially Decoded image dataset constructed by retaining exactly *x* quantized residual coefficients via the LFSP method, while Px-Vanilla represents the standard, fully decoded pixel-domain baseline. Regarding the performance metrics across the tables, Bitrate indicates the average volume of the transmitted bitstream; Dtime represents the processing latency isolated to the decoding stage; Inference is the neural network execution time; and Total represents the end-to-end pipeline latency which is the sum of Dtime and Inference. Furthermore, detection accuracy is measured by mAP and ${\mathrm{mAP}}_{S,M,L}$ which represent the mean Average Precision specifically for various scales of objects.

Table 5 illustrates the computational and bandwidth efficiency of the varying LFSP configurations in the same experimental settings. As fewer coefficients are retained, the decoding time and bitrate drop significantly. Table 6 incorporates the best accuracy metrics over numerous experiments for the respective configurations to understand the trade-off between these computational gains and detection fidelity. As the number of retained coefficients increases from PD1 to PD100, we again observe a clear trajectory of diminishing returns. Furthermore, examining the image quality metrics (SSIM and PSNR) in Table 4 reveal that the performance gain associated with incrementing the retained residual coefficients is highly intuitive.

In short, the PD64 configuration emerges as the optimal operational threshold for the framework based on this comprehensive analysis. Concurrently, the PD64 configuration maintains a highly efficient decoding latency of 13.9 ms, bringing the total processing time down to 68.72 ms. This demonstrates a substantial improvement over the baseline’s 73.93 ms. Beyond the 64-coefficient threshold, incorporating additional high-frequency spectral data introduces a severe escalation in latency with negligible semantic benefit. Expanding to PD81 and PD100 increases the decoding time to 14.9 ms and 15.4 ms, respectively, driving the total pipeline latency steadily closer to the standard fully decoded baseline. Conversely, the corresponding gains in detection accuracy become virtually stagnant; overall mAP increases by merely 0.4% and 0.6% relative to PD64, while ${\mathrm{mAP}}_{L}$ also increases only marginally. In this case the minimal objective quality improvements beyond 64 coefficients do not justify the disproportionate escalation in decoding time and transmission bandwidth.

To rigorously validate the proposed compressed-domain distillation framework and address the comprehensive performance metrics raised, we present an expanded evaluation in Table 7 and Table 8. To ensure a strictly fair comparison between the partially decoded (PD64) compressed-domain student and the fully decoded pixel-domain (Px) baseline, all models were evaluated under identical conditions. Specifically, we enforce identical training settings, such as learning rate (0.01), batch size (8), and maximum epochs (20), alongside identical test-time augmentation and matching input preprocessing and augmentation pipelines. Furthermore, student and vanilla baseline model capacities are held constant across all benchmarked configurations to isolate the effects of our method with given teacher model capacities. To provide robust statistical validation and address potential run-to-run variance, all results reported in Table 7, Table 8, Table 9 and Table 10 are presented as the mean and standard deviation derived from five independent experimental runs. This confirms that the performance gains represent statistically significant and stable improvements with clear confidence intervals. As can be seen from Table 7, the KD-enhanced student network achieves an overall mAP of 25.08% and successfully surpasses the fully decoded Px-Vanilla baseline performance mAP of 24.12%.

While our multi-granularity cross-domain knowledge distillation framework yields significant accuracy improvements, it introduces additional computational overhead during the training phase. Specifically, maintaining both the teacher and student networks alongside the distillation branches (GFD, FBFD, LRD) increases the total training time from 7 h to 20 h, and raises the peak GPU memory utilization from 2.67 GB to 5.95 GB. It is important to note, however, that this represents a one-time offline cost. During actual deployment, the teacher network and distillation mechanisms are completely discarded, ensuring that the inference phase retains its stringent efficiency and low latency footprint.

Directly comparing our method against previous compressed-domain object detection architectures is challenging, primarily because our framework introduces a unique paradigm combining partial decoding with cross-domain knowledge distillation. However, to explicitly demonstrate the progressiveness of our methodology, we integrated the recent compressed-domain strategy proposed in [30], denoted as RP, into our evaluation framework. As shown in Table 7, our PD64 configuration substantially outperforms the RP method. Using the same R50-to-R18 teacher–student distillation architecture, PD64 achieves a mAP of 25.08% compared to 10.12%. Furthermore, PD64 processes at 68.72 ms, maintaining highly competitive end-to-end latency while delivering a substantial accuracy gain.

An essential aspect of compressed-domain detection is understanding how partial decoding affects features at different spatial resolutions. PD64 exhibits significant strength in detecting large objects and achieves a ${\mathrm{mAP}}_{L}$ of 38.44% compared to 32.64% against the fully decoded Px-Vanilla baseline in Table 7. Large objects are inherently less affected by the truncation artifacts introduced during partial decoding, which allows the network to effectively leverage the rich, uncorrupted semantic cues retained in the compressed domain. Performance on medium objects remains highly robust, with PD64 gracefully outperforming the baseline with a ${\mathrm{mAP}}_{M}$ of 26.20% compared to 25.48%. Conversely, small object detection presents a known challenge in compressed-domain networks. Our PD64 method yields a ${\mathrm{mAP}}_{S}$ of 8.28%, which is slightly lower than baseline’s 10.96%. Small objects rely heavily on high-frequency spatial details which are naturally attenuated or lost during partial decoding.

To substantiate the claim that our cross-domain knowledge distillation does not depend on a single architecture pairing, we tested an additional backbone pairing to observe the effects of a reduced teacher–student capacity gap. Table 7 presents the results utilizing a ResNet-34 (R34) teacher alongside the ResNet-50 (R50) teacher. The R34-to-R18 configuration yields mAP of 24.82%, proving that our method is generalizable and not strictly reliant on massive capacity disparities to successfully transfer compressed-domain representations. Finally, we extended our rigorous evaluation to the complete COCO dataset in Table 8. The PD64 student reliably surpasses the vanilla Px baseline on this large-scale dataset and achieves an overall mAP of 33.69% compared to 32.44%. The improvement remains most pronounced in large object detection, where PD64 reaches a ${\mathrm{mAP}}_{L}$ of 51.13% against the baseline’s 44.44%. Crucially, this superior accuracy is coupled with a distinct efficiency advantage, as the PD64 pipeline registers a faster total processing time of 63.53 ms compared to the fully decoded baseline’s 68.74 ms.

To further demonstrate the generalizability and architectural robustness of the proposed method, we extended our evaluation beyond the RetinaNet to include two other object detectors: Fully Convolutional One-Stage Object Detection (FCOS) [49] and Generalized Focal Loss (GFL) [50]. For these experiments, we maintained the established cross-domain knowledge distillation architecture, utilizing a high-capacity ResNet-50 teacher operating in the pixel domain to guide a lightweight ResNet-18 student. Based on our comprehensive analysis of the RetinaNet results, retaining exactly 64 quantized residual coefficients emerged as the optimal operational threshold. Consequently, we exclusively utilized PD64 generated via LFSP residual removal method for this phase of the evaluation.

Table 9 quantitatively summarizes the computational and latency metrics for the FCOS and GFL detectors operating on fully decoded pixel-domain inputs (Px-Vanilla) versus the partially decoded inputs (PD64). Consistent with our earlier observations, the empirical results reveal compelling advantages of our method in computational efficiency across both alternative architectures. The implementation of the LFSP residual removal method successfully short-circuits the standard decoding pipeline and this decoding acceleration translates directly to the end-to-end pipeline. The total processing time is reduced by approximately 5.2 ms for both the FCOS and GFL detectors.

To understand the detection performance of the proposed method, Table 10 presents the end-to-end detection accuracy metrics over 5 experiments for the respective configurations. Remarkably, the compressed-domain detection actively surpasses the baseline pixel-domain performance, despite operating on relatively small bitstreams. For the FCOS detector, the PD64 configuration elevates the overall mAP from 16.44% to 18.58%, while the GFL detector’s results show an overall mAP rise from 25.52% to 25.85%.

### 5.4. Ablation Study on Knowledge Distillation Components

To rigorously quantify the individual and synergistic contributions of the various knowledge distillation mechanisms utilized within our framework, we conducted a comprehensive ablation study. For this analysis, we maintained the optimal configuration established in the preceding experiments: the RetinaNet detector featuring a ResNet-18 student network guided by a ResNet-50 teacher network and operating exclusively on PD64 generated via the LFSP partial decoding method. The quantitative results of this ablation study are presented in Table 11 with best accuracy metrics over numerous experiments. To systematically evaluate these contributions, our ablation logic proceeds in three progressive stages: first, we analyze the standalone performance of each individual distillation method; second, we examine the dual performance achieved by pairing methods; and finally, we assess the performance resulting from the full integration of all components.

When evaluated in isolation during the first stage, Global Feature Distillation emerges as the most impactful standalone branch. It achieves an overall mAP of 23.41% and a ${\mathrm{mAP}}_{L}$ of 35.13%. This high performance underscores its ability to extract hierarchical features from the entire image area and effectively forces the student model to maintain a coherent understanding of the global spatial structure. Foreground–Background Feature Distillation also demonstrates strong independent performance. By utilizing binary masks to decouple foreground targets from background features, it successfully prevents the student network from being distracted by the compression artifacts that frequently corrupt background regions. In contrast, relying solely on Logit Response Distillation yields the lowest performance. This indicates that simply forcing the student to mimic the teacher’s class-probability distribution is highly inefficient when the underlying feature representations are not properly aligned to handle the severe domain gap.

In the subsequent stage evaluating dual performance, the integration of both feature-level distillations yields a substantial boost that raises mAP to 24.32%. This combination ensures that the student simultaneously captures the broad scene context while cleanly distinguishing structural target from ambient noise.

Finally, assessing the combined performance through the full integration of all three distillation branches achieves the peak performance of the framework. It maximizes the overall mAP at 25.30% and ${\mathrm{mAP}}_{L}$ to 38.70%. We hypothesize that once the Global and FGBG distillations establish a robust, artifact-resistant feature foundation, the Logit distillation can effectively operate on these aligned representations. This complete configuration proves exceptionally capable of bridging the pixel-to-compressed-domain gap and validates our approach of extracting highly reliable semantic inferences from partially decoded streams.

## 6. Conclusions

In this study, we addressed the critical computational bottlenecks inherent to standard video analytics on resource-constrained edge computation by proposing a fast and efficient object detection framework operating in the compressed domain. We introduce a partial decoding method which utilizes residual removal strategy. By implementing LFSP at the encoder, our framework systematically truncates non-essential residual coefficients. This strategic omission generates a highly compact bitstream, naturally accelerates the standard decoding process by bypassing exhaustive computations, and yields a highly efficient partially decoded representation. To bridge the semantic gap and mitigate the accuracy degradation, we developed a cross-domain knowledge distillation strategy. Extensive empirical evaluations on both the COCO-mini and the complete COCO datasets confirm that retaining exactly 64 quantized residual coefficients yields an optimal balance, and enables the distillation-enhanced student networks to surpass fully decoded baselines. Despite these promising results, our current methodology is limited to intra-frame evaluation and has not yet been physically deployed on edge hardware. However, practical deployment can be easily realized by implementing the necessary modifications exclusively at the camera side in the future; this eliminates intensive processing requirements at the destination. Thus, the small and lightweight compressed-domain object detection network can operate on standard edge processors. To further advance this approach, future research will focus on extending the framework to inter-frame video streams by incorporating the method on P and B frames, as well as exploring compatibility with implementing the method on the other legacy codecs and modern standards such as VVC.

## Figures and Tables

**Table 1:** Summary of methods for object detection in compressed domain (MOD: Moving Object Detection, OD: Object Detection, LPD: License Plate Detection, VOS: Video Object Segmentation, DOD: Dense Object Detection, CDKD: Cross-Domain Knowledge Distillation).

Work	Focus/Method	Task	CDKD
Laumer et al. (2016) [28]	Spatiotemporal segmentation evaluating H.264/AVC macroblock types, partition modes, and QPs	MOD	No
Zhao et al. (2016) [6]	Segmentation and classification using a bag of spatio-temporal HEVC syntax words	MOD	No
Jaballah et al. (2019) [29]	Object segmentation via spatio-temporal median filtering and fuzzy clustering of coding parameters	MOD	No
Wang et al. (2019) [33]	Fast detection via Motion-aided Memory Network (MMNet) with pyramidal feature attention	OD	No
Beratoğlu et al. (2021) [7]	License plate detection using block partition and prediction unit images from HEVC intra-frames	LPD	No
Chen et al. (2021) [12]	Fast detection in HEVC intra-frames via an iterative residual restoration algorithm	OD	No
Tan et al. (2021) [34]	Feature warping with residual supplement and spatial attention	VOS	No
Feng et al. (2022) [35]	TapLab semantic video segmentation utilizing motion vectors and residuals	DOD	No
Dror et al. (2025) [31]	Dense detection by dynamically categorizing CTUs into ROIs	DOD	No
Beratoğlu et al. (2023) [30]	Vehicle detection/classification via residual-free image reconstruction using random perturbations	OD	No
Zain et al. (2026) [32]	Detection via adaptive selective encryption on base layers	OD	No

**Table 2:** Summary of methods for knowledge distillation from compressed domain. Abbreviations AR: Action Recognition, SS: Semantic Segmentation, OD: Object Detection, C: Classification, LPD: License Plate Detection, PD: Partial Decoding.

Work	Focus/Methods	Task	PD
Zhang et al. (2016) [36]	Knowledge transfer from optical flow to motion vector CNNs via initialization and supervision transfer	AR	Yes
Huang et al. (2019) [37]	Generalized distillation from optical flow to enhance joint motion vector and residual streams	AR	Yes
Lu et al. (2021) [18]	Boundary-aware stream guiding features combined with pixel-wise and inner-relation knowledge distillation	SS	No
Huo et al. (2020) [38]	Feature alignment distillation between optical flow and MV networks coupled with temporal trilinear pooling	AR	Yes
Li et al. (2021) [39]	Joint feature optimization with self-adaptive knowledge distillation to mimic optical flow-based predictions	AR	Yes
Liu et al. (2023) [17]	Cross-modality distillation using pseudo-decoders for spatial knowledge and temporal graphs for temporal knowledge	AR	Yes
Duan et al. (2023) [40]	Pixel-to-compressed-domain knowledge transfer minimizing intermediate feature map discrepancies	SS	No
Liu et al. (2022) [41]	MSE distillation of middle-layer features combined with adaptive channel-selection gate modules	OD/SS	No
Keser et al. (2024) [42]	Cross distillation transferring compressed-domain (HEVC) hints to enhance a pixel-domain student	C/LPD	Yes

**Table 3:** Quantitative comparison of partial decoding configurations.

Methodology	Dec. Time (ms)	Bit Size (KB)	PSNR (dB)	SSIM
Fully Decoded	19.10	18.46	35.90	0.9968
SRSP	12.80	9.48	18.54	0.7479
LFSP	13.90	11.25	26.57	0.8491

**Table 4:** Scalability and performance metrics of LFSP over varying retained quantized residual coefficients.

Residual Coeff.	Dec. Time (ms)	Size (KB)	PSNR (dB)	SSIM
1	10.8	2.89	15.10	0.5901
9	10.5	4.57	14.12	0.6214
25	11.9	7.62	21.90	0.7634
36	12.5	8.71	22.46	0.7813
49	13.3	10.27	23.18	0.8022
64	13.9	11.25	26.57	0.8491
81	14.9	12.64	27.31	0.8604
100	15.4	13.40	27.86	0.8685

**Table 5:** Computational latency metrics across LFSP configurations.

Img Type	Bitrate [kB]	Dtime [ms]	Inference [ms]	Total [ms]
PD1	2.89	10.8	54.81	65.61
PD9	4.57	11.4	54.83	66.23
PD25	7.62	11.9	54.81	66.71
PD36	8.71	12.5	54.83	67.33
PD49	10.27	13.3	54.82	68.12
**PD64**	**11.25**	**13.9**	**54.82**	**68.72**
PD81	12.64	14.9	54.84	69.74
PD100	13.40	15.4	54.83	70.23
Px-Vanilla	18.46	19.1	54.83	73.93

**Bold text** indicates the optimal operational threshold for the framework.

**Table 6:** End-to-End performance and accuracy comparison of RetinaNet on COCO-mini dataset. (R50-Teacher, R18-Student).

Img Type	Total [ms]	mAP [%]	${\mathrm{mAP}}_{L}$ [%]
PD1	65.61	7.6	14.8
PD9	66.23	12.7	23.0
PD25	66.71	20.8	34.0
PD36	67.33	22.0	36.1
PD49	68.12	23.1	36.4
**PD64**	**68.72**	**25.3**	**38.7**
PD81	69.74	25.7	38.9
PD100	70.23	25.9	39.0

**Bold text** indicates the optimal operational threshold for the framework.

**Table 7:** End-to-End performance and accuracy comparison of RetinaNet over 5 runs on COCO-mini dataset. Both mean (*m*) and standard deviation (std) of all mAP scores are reported in the table in following notation: m±std.

Img Type	Teacher Backbone	Student Backbone	Total [ms]	mAP [%]	${\mathrm{mAP}}_{L}$ [%]	${\mathrm{mAP}}_{M}$ [%]	${\mathrm{mAP}}_{S}$ [%]
RP [30]	R50	R18	65.32	10.12±0.15	18.96±0.40	8.02±0.13	1.76±0.05
PD64	R34	R18	68.72	24.82±0.13	37.98±0.22	25.90±0.20	7.76±0.15
PD64	R50	R18	68.72	25.08±0.15	38.44±0.23	26.20±0.36	8.28±0.22
Px	Vanilla	R18	73.93	24.12±0.19	32.64±0.39	25.48±0.29	10.96±0.23

**Table 8:** End-to-End performance and accuracy comparison of RetinaNet over 5 runs on the complete COCO dataset. Both mean (*m*) and standard deviation (std) of all mAP scores are reported in the table in following notation: m±std.

Img Type	Teacher Backbone	Student Backbone	Total [ms]	mAP [%]	${\mathrm{mAP}}_{L}$ [%]	${\mathrm{mAP}}_{M}$ [%]	${\mathrm{mAP}}_{S}$ [%]
Px	Vanilla	R18	68.74	32.44±0.24	44.44±0.43	35.15±0.34	17.24±0.19
PD64	R50	R18	63.53	33.69±0.13	51.13±0.21	36.55±0.19	12.66±0.15

**Table 9:** Computational latency comparison of FCOS and GFL detectors (R50: Teacher; R18: Student).

Detector	Img Type	Dec. Time [ms]	Inference [ms]	Total [ms]
FCOS-Vanilla	Px	19.1	49.64	68.74
FCOS-PD	PD64	13.9	49.63	63.53
GFL-Vanilla	Px	19.1	48.83	67.93
GFL-PD	PD64	13.9	48.81	62.71

**Table 10:** End-to-End accuracy comparison of FCOS and GFL detectors over 5 runs on COCO-mini dataset (R50: teacher; R18: student). Both mean (*m*) and standard deviation (std) of all mAP scores are reported in the table in following notation: m±std.

Detector	Img Type	Total [ms]	mAP [%]	${\mathrm{mAP}}_{L}$ [%]	${\mathrm{mAP}}_{M}$ [%]	${\mathrm{mAP}}_{S}$ [%]
FCOS-Vanilla	Px	68.74	16.44±0.17	21.68±0.30	17.78±0.22	8.80±0.38
FCOS-PD	PD64	63.53	18.58±0.05	27.15±0.44	20.25±0.17	7.18±0.19
GFL-Vanilla	Px	67.93	25.52±0.11	34.46±0.39	26.30±0.16	12.33±0.24
GFL-PD	PD64	62.71	25.85±0.10	38.58±0.43	27.55±0.12	9.87±0.20

**Table 11:** Ablation study of knowledge distillation components on RetinaNet (PD64 configuration).

Global	FGBG	Logit	mAP [%]	${\mathrm{mAP}}_{L}$ [%]
✓	×	×	23.41	35.13
×	✓	×	22.95	34.39
×	×	✓	19.89	29.52
✓	×	✓	22.16	33.54
✓	✓	×	24.32	37.35
×	✓	✓	24.20	37.14
**✓**	**✓**	**✓**	**25.30**	**38.70**

The ✓ and × symbols denote the inclusion and exclusion of the respective knowledge distillation branch. **Bold text** indicates the optimal configuration and the best performance metrics.

## Data Availability

The COCO dataset used in this study is publicly available at https://cocodataset.org/#download (accessed on 13 July 2026). The datasets generated during the current study are available from the corresponding author upon request. The source code is publicly available at https://github.com/sdikyar/EffODinCDwKD (accessed on 13 July 2026).

## Abbreviations

The following abbreviations are used in this manuscript:

AVC	Advanced Video Coding
HEVC	High-Efficiency Video Coding
VVC	Versatile Video Coding
GDPR	General Data Protection Regulation
COCO	Common Objects in Context
FCOS	Fully Convolutional One-Stage Object Detection
GFL	Generalized Focal Loss
MV	Motion Vector
DCT	Discrete Cosine Transform
CU	Coding Unit
CTU	Coding Tree Unit
SRSP	Simple Raster Sequence Prioritization
LFSP	Low-Frequency Spectral Prioritization
GFD	Global Feature Distillation
FBFD	Foreground–Background Feature Distillation
LRD	Logit-based Response Distillation
KL	Kullback–Leibler
PD	Partial Decode
PSNR	Peak Signal-to-Noise Ratio
SSIM	Structural Similarity
mAP	Mean Average Precision
${\mathrm{mAP}}_{L}$	Mean Average Precision for Large Objects
${\mathrm{mAP}}_{M}$	Mean Average Precision for Medium Objects
${\mathrm{mAP}}_{S}$	Mean Average Precision for Small Objects
